# Prevalence of dengue and diversity of cultivable bacteria in vector *Aedes aegypti* (L.) from two dengue endemic districts, Kanchanpur and Parsa of Nepal

**DOI:** 10.1186/s41043-017-0080-6

**Published:** 2017-02-13

**Authors:** Srinivas Thapa, Narayan Dutt Pant, Rojina Shrestha, Ganga GC, Bidya Shrestha, Basu Dev Pandey, Ishan Gautam

**Affiliations:** 1 Department of Microbiology, Tri-Chandra Multiple Campus, Kathmandu, Nepal; 2 Department of Microbiology, Grande International Hospital, Dhapasi, Kathmandu, Nepal; 3 Everest International Clinic and Research Center, Kathmandu, Nepal; 4 Institute of science and technology, Tribhuvan University, Natural History Museum, Kathmandu, Nepal

**Keywords:** Dengue, *Aedes aegypti*, Paratransgenesis, Nepal

## Abstract

**Background:**

Dengue fever, an endemic arboviral disease, represents one of the major public health concerns in Nepal. It is transmitted by bites of infected *Aedes aegypti* and *Aedes albopictus*, the former being primary vector. The bacterial community plays a significant role in biology of mosquitoes; however, the bacterial communities of primary vector *A. aegypti* remain unstudied in Nepal. The study was designed to determine the rate of dengue seropositivity and to explore the bacterial diversity of *A. aegypti* from dengue endemic districts, Kanchanpur and Parsa of Nepal.

**Methods:**

A cross-sectional study was conducted between June 2013 and November 2013 at two hospitals of Kanchanpur and Parsa. A total of 221 serum samples were collected from patients (inpatients and outpatients) suspected of suffering from dengue fever and attending Mahakali Zonal Hospital, Mahendranagar, Kanchanpur, and Narayani Zonal Hospital, Birgunj, Parsa. Detection of anti-dengue IgM was performed by using human dengue IgM capture ELISA. The larvae and pupae of mosquitoes (*A. aegypti*) were collected, reared, and emerged. Then, the bacteria were isolated and identified from the gut of identified mosquitoes by using standard methods.

**Results:**

Out of total 221 serum samples collected from patients suspected of suffering from dengue fever, 34 (15.38%) were positive for anti-dengue IgM. Gram-negative bacteria were isolated in largest proportion (63%) followed by gram-positive cocci (23.27%) and gram-positive rods (13.73%). The most common cultivable bacteria isolated were *Staphylococcus* spp*.*, *Pseudomonas* spp., and *Acinetobacter* spp. The average bacterial load in the vectors was 3.91 × 10^4^ cfu/ml.

**Conclusions:**

High rate of anti-dengue IgM seropositivity was reported in our study. The environmental bacteria were predominantly isolated and identified in *A. aegypti*. The paratransgenic approach to control vector might be possible by spreading the genetically modified bacteria in larval habitat or shelter of adult mosquitoes.

## Background

Vector-borne diseases are serious public health problems, accounting for more than 17% of all infectious diseases, causing more than 1 million deaths annually [1]. Among them, dengue causes 390 million infections per year with clinical symptoms of variable severity in 96 million of population [2]. In Southeast Asia and Western Pacific region, approximately 1.8 billion residents are at the risk of getting infected with dengue virus [3]. There have been reports of dengue fever (DF)/dengue hemorrhagic fever (DHF) epidemics in Bhutan, India, Maldives, Bangladesh, and Pakistan, and due to porous borders with India there is high risk of DF/DHF outbreaks in Nepal [3]. Further, during past decade significant morbidity and mortality due to dengue fever have been reported from different districts of Terai region of Nepal [3]. Since the first case of dengue reported in Nepal in 2004, different prevalences of dengue have been reported by different authors (8.99–30%) in Nepal [3].

Effective vector control measures may indeed contribute to reduce morbidity and mortality attributable to dengue but emergence of insecticide resistance in vectors and issue of environmental pollution suggest the need of change in vector control practices [4]. Paratransgenesis is an eco-friendly biological method for vector control, focuses on exploiting insect symbionts genetically to express molecules within the vectors, which causes deleterious effect to pathogens they transmit [5]. But the composition of mosquito-associated microbial communities should be known prior to development of the paratransgenesis.

Despite the significant morbidity and mortality due to dengue in Nepal, as a part of effective and environment friendly vector control program, mosquito-associated microbial communities are unstudied in context of Nepal and their potential effect on host life is unrevealed. It has been known that the bacteria belonging to *Enterobacter* spp., *Klebsiella* spp., *Pseudomonas* spp., *Serratia* spp., *Acinetobacter* spp., *Asaia* spp., *Bacillus* spp., *Staphylococcus* spp., *Comamonas* spp., *Delfti* spp., and *Wolbachia* spp. occur as normal flora in *Aedes aegypti* or *Aedes albopictus* [6–10]. Microbial symbionts of *A. aegypti* significantly influence the vector competence, the measurement of genetic ability of insects to transmit pathogens [11, 12].

In this study, we determined the rate of anti-dengue IgM seropositivity among the patients suspected of suffering from dengue fever. Further, we isolated and identified the cultivable microbiota from gut of vector *A. aegypti*. These flora can be exploited for vector control, and this will not only reduce the problem resulting from emergence of insecticide resistance in vectors but also help in controlling of environment pollution due to use of insecticides.

## Methods

A descriptive cross-sectional study was conducted from June 2013 to November 2013 at two hospitals of two dengue endemic districts, Kanchanpur, and Parsa of Nepal. The sample sizes were calculated by using the formula:

$$ \mathrm{Sample}\ \mathrm{size}={\mathrm{Z}}^2\mathrm{P}\left(1- P\right)/{C}^2 $$

Where,

*Z* = *Z* value (1.96 for 95% confidence level)

*P* = prevalence

*C* = confidence interval (0.05)

The prevalence of dengue was found to be reported around 15% by different studies in developing countries including Nepal. Similarly, the container index was found to be reported around 10%. So, the sample size was calculated to be approximately 196 for patients and 138 for wet containers. However, we take higher numbers of samples.

A total of 221 (121 from inpatients and 100 from outpatients) blood samples were collected after 6 to 10 days after onset of illness from all the patients (inpatients and outpatients) suspected of suffering from dengue fever (having complaints of febrile illness with two or more symptoms like ocular pain, headache, rash, and arthralgia or myalgia for around 1 week) and attending Mahakali Zonal Hospital, Mahendranagar, Kanchanpur, and Narayani Zonal Hospital, Birgunj, Parsa during the study period. The 3 to 5 ml of blood sample was collected (by venipuncture by using aseptic technique) from each patient by the trained health care worker, and the serum was then extracted from the blood. The serum samples were transported to laboratory in Kathmandu maintaining cold chain.

These two hospitals where we conducted the research were the referral centers for all the cases of suspected dengue fever from their respective districts, and the cases of suspected dengue fever were referred to those hospitals from other hospitals from all over the districts of respective hospitals. IgM antibody is the first immunoglobulin isotype to appear in an infection. So, to diagnose current infection, detection of anti-dengue IgM was performed by using human dengue IgM capture ELISA (Human GeseellschaftfÜr biochemical and diagnostic mbH, Germany) at Everest International Clinic and Research Center, Kathmandu, Nepal.

The potential habitats present in the residential area were searched for the presence of immature dengue vectors. Total 166 wet containers (83 from each district) of different types (discarded tires, plastic buckets, metal drums, roofs, cemented tanks, plastic tanks, plastic bottles, and earthen pots) were examined. The whole districts were divided into 10 approximately equal parts. Within each part, a main residential area was located and the areas were thoroughly searched for the potential vector breeding sites until we collected 8 wet containers from each 7 locations and 9 from remaining 3 larger locations. The wet containers those can hold large amount of water with a preferable environment (e.g., discarded tires, plastic buckets, metal drums) for mosquitoes were preferably sampled. To find the exact location of the mosquito breeding and the correlation of the cultivable bacterial concentration in gut of the mosquito with the surrounding of the habitat, larvae, and pupae were collected rather than capturing adult mosquitoes, as the adult mosquitoes may migrate to other locations after emerging somewhere else. The larvae and pupae of mosquitoes were collected by dropper and dipper method. Then they were reared for emergence of adult mosquitoes at Natural History Museum, Kathmandu [13]. Based on the different morphological characteristics, the adult mosquitoes were identified by using taxonomic keys published by Darsie and Pradhan [14]. The mosquitoes identified as female *A. aegypti* were anesthetized using chloroform and rinsed 3 times in sterilized water. Then they were surface sterilized in 70% ethanol for 5 min, rinsed 3 times in sterilized water, and once in sterilized 0.8% sodium chloride solution. The viscera of mosquitoes were extracted in test tube with 2 ml of sterilized 0.8% sodium chloride solution. The resulting solutions were serially diluted, and the 0.1 ml of each solution was inoculated on each chocolate agar, blood agar, and nutrient agar by spread plate technique and incubated at room temperature for 72 h [9, 15, 16].

The bacterial concentration was determined in terms of cfu/ml, and the bacteria grown were identified by using standard microbiological techniques as described in the Bergey’s Manual of Systematic Bacteriology [17]. For data analysis, SPSS version 16.0 was used. Chi-square test was applied and *p* value < 0.05 was taken as statistically significant.

## Results

### Serological test

Out of total 221 (126 from males and 95 from females) serum samples from patients suspected of suffering from dengue fever, 34 (15.38%) were positive for anti-dengue IgM.

### Gender and age-wise distribution of seropositive dengue cases

The rate of seropositivity was 19.05% (24/126) in case of males and 10.53% (10/95) in case of females. The difference was statistically insignificant. The rate of dengue seropositivity was highest in age group 31–45 years (25%) followed by 15 to 30 years (19.77%) (*p* < 0.05) (Table 1).

**Table 1:** Age-wise distribution of seropositive dengue cases

Age	Total no. of samples	No. of positive samples (%)	*p* value
<15 years	49	2 (4.08)	
15–30 years	86	17 (19.77)	
31–45 years	40	10 (25)	
46–60 years	28	2 (7.14)	0.034
>60 years	18	3 (16.67)	
Total	221	34	

### Study site-wise distribution of dengue cases

Rate of dengue seropositivity was 23.28% (27/116) among the samples collected from Narayani Zonal Hospital, Birgunj, Parsa, while that was 6.67% (7/105) among samples collected from Mahakali Zonal Hospital, Mahendranagar, Kanchanpur.

### Bacterial loads in vector, *A. aegypti* according to habitats

The bacterial loads in the vector ranged from 3.13 × 10^4^ cfu/ml to 4.71 × 10^4^ cfu/ml with highest load being in vector collected from discarded tires found around the timber trees. The average bacterial count in vectors was 3.91 × 10^4^ cfu/ml (Table 2).

**Table 2:** Bacterial loads in vector *Aedes aegypti* according to habitats

Habitats	Zone	Vegetation around habitats	cfu/ml
Discarded tires	City	Bushes	4.18 × 10^4^
	City outskirt	Timber trees	4.71 × 10^4^
Cemented tanks	City	Fruits trees	3.87 × 10^4^
	City outskirt	Timber trees	4.23 × 10^4^
Plastic buckets	City	Bushes	3.98 × 10^4^
	City outskirt	Timber trees	4.03 × 10^4^
Metal drums	City	Fruits trees	3.72 × 10^4^
	City outskirt	Bushes	3.13 × 10^4^
Average cfu/ml			3.91 × 10^4^

### Culturable microbiota of dengue vector, *A. aegypti*

Gram negative bacteria were isolated in largest proportion (63%) followed by gram positive cocci (23.27%), and gram positive rods (13.73%). Based on colony morphology, gram stain reaction and conventional biochemical properties, 64% of the isolated colonies were identified and 36% remained unidentified. Among identified colonies, 33.28% were *Staphylococcus* spp*.* followed by *Pseudomonas* spp. (29.42%), *Acinetobacter* spp. (22.05%), and *Bacillus* spp. (15.25%). In bacterial diversity analysis, Shannon Weaver diversity index (H) and Evenness (E) were found to be 1.34 and 0.97, respectively.

## Discussion

The prevalence of anti-dengue IgM positivity reported in our study was in accordance with the findings by Shrestha et al. (19.31%) [3] and Poudel et al. (12.17%) [18]. However, higher rates of positivity were reported by Sah et al. (30%) [19] and Gupta et al. (29.09%) [20], whereas lower rates were reported by Shah et al. (8.99%) [21] and Shah et al. (9.8%) [22]. The variation in the results reported by different studies from different places during different periods of time might be due to geographical variation, difference in climate that directly influence the vector population, and difference in effectiveness of vector control programs [3]. As in the study by Gupta et al. [23], Poudel et al. [18], and Gupta et al. [20], we reported higher rate of dengue seropositivity in males in comparison to females, but the difference was statistically insignificant. However, higher rates were reported in females by Shah et al. [22] and Shrestha et al. [3]. In present study, the rate of dengue seropositivity was found to be highest in age group 30 to 45 years followed by 15 to 30 years and which was statistically significant and against the popular belief that dengue is a pediatric disease [24]. This might be due to higher rate of exposure to the dengue vector in these dengue endemic districts among the people of this age group, as this age group is most active age group taking part in outdoor activities like cattle grazing, farming, searching for firewoods, and grasses in jungles.

In a study conducted in 2010, Gupta et al. [23] reported the rate of dengue seropositivity to be 5.52% in Birgunj, which was 23.28% in our study. Similarly, during 2008/2009, Shah et al. [22] showed the prevalence of dengue seropositivity in Mahendranagar to be 13.3% which was 6.67% in our study. The comparatively higher positivity rate in Birgunj might be due to higher endemicity of the disease in the area. Further, the sanitation facilities of Birgunj are poorer in comparison to those in Mahendranagar, which contribute to the fertile breeding grounds for mosquitoes like *Aedes* spp. Birgunj and Mahendranagar both are open bordered with northern part of India which is a dengue endemic region.

The isolation and enumeration of bacteria in vector *A. aegypti* was performed by spread plate technique. Highest bacterial load (4.71 × 10^4^ cfu/ml) was detected in vectors collected from discarded tires found around timber trees. This might be due to the fact that shelter provided by tree made suitable environment for bacterial growth. The debris produced from tree also helps to flourish the bacterial growth. As the bacteria are sources of food for larvae of mosquitoes, the bacterial load of habitats influences the bacterial load in mosquitoes. The average bacterial load in vector *A. aegypti* was found to be 3.91 × 10^4^ cfu/ml. In our study, the largest proportion of the bacteria isolated were gram-negative bacilli (63%), which was in harmony with the previous studies by Apte-Deshpande et al. (64%) [16], Ramirez et al. (85%) [25], and Valiente Moro et al. (51.3%) [10].

Some bacteria presenting as normal flora of mid gut of mosquitoes contribute significantly in disease transmission, host-parasites interaction, and determination of vectorial capacity [26]. In paratransgenesis, symbiotic bacteria are genetically exploited and introduced into the vectors, where the bacteria may act either by causing deleterious effect to pathogens transmitted by the vector or by disturbing the reproduction or making the vector less competent or reducing the life span of the host [27]. However, the better understanding of the composition of mosquito-associated microbial communities is necessary prior to development of this approach [27]. *Enterobacter*, *Bacillus*, *Pseudomonas*, *Staphylococcus*, *Klebsiella*, *Pantoea*, *Acinetobacter*, and *Aeromonas* have been identified as the normal flora of mid gut of mosquitoes and our findings were in accordance with the results reported by other researchers [26]. The interaction with environment in different stages of life cycle might play a significant role in colonization of mosquitoes by bacteria present in the environment [28]. *Acinetobacter* which was one of the predominant isolates in our study is known to be helpful to mosquitoes for blood digestion [26]. So the bacteria which are known to be actively involved in chemical and biological processes in the insect host can be identified and novel bacterial genes responsible for these processes can be explored; so that the knowledge can be used for vector control by paratransgenesis [28].

In our study, the Shannon Weaver index (H) and Evenness (E) in *A. aegypti* were found to be 1.34 and 0.97, respectively. The results were in accordance with previous study conducted by Zouche et al., where H was 1.16–2.45 and E was 0.80–0.86 [29]. The numeric values of diversity indices H and E suggested the higher diversity of bacterial community.

### Limitations of the study

Among isolated bacteria, small proportion was remained unidentified, due to lack of advance laboratory facility. Further, we could not use molecular techniques to identify the uncultivable bacteria (which are the important part of normal flora of mosquito) found in mosquito and to confirm our results.

## Conclusions

High rate of anti-dengue IgM seropositivity was reported in our study. The rate was higher in Birgunj, Parsa in comparison to Mahendranagar, Kanchanpur. The study of microbial diversity in *A. aegypti* revealed that environmental bacteria were dominantly present. The paratrangenic approach to control vector might be possible by spreading the genetically modified bacteria in larval habitat or shelter of adult mosquitoes. Among all the bacteria isolated *Acinetobacter* which was one of the predominant bacterium isolated in our study and is known to be involved in important metabolic activity in mosquitoes may be an important option for using in paratransgenesis.

## Abbreviations

DF: Dengue fever
DHF: Dengue hemorrhagic fever
ELISA: Enzyme-linked immunosorbent assay
SPSS: Statistical Package for the Social Sciences
